# *GmDREB1* overexpression affects the expression of microRNAs in GM wheat seeds

**DOI:** 10.1371/journal.pone.0175924

**Published:** 2017-05-01

**Authors:** Qiyan Jiang, Xianjun Sun, Fengjuan Niu, Zheng Hu, Rui Chen, Hui Zhang

**Affiliations:** 1 Institute of Crop Science, National Key Facility for Crop Gene Resources and Genetic Improvement, Chinese Academy of Agricultural Sciences, Beijing, China; 2 Tianjin Institute of Agricultural Quality Standard and Testing Technology, Tianjin Academy of Agricultural Sciences, Tianjin, China; Dokuz Eylul Universitesi, TURKEY

## Abstract

MicroRNAs (miRNAs) are small regulators of gene expression that act on many different molecular and biochemical processes in eukaryotes. To date, miRNAs have not been considered in the current evaluation system for GM crops. In this study, small RNAs from the dry seeds of a GM wheat line overexpressing *GmDREB1* and non-GM wheat cultivars were investigated using deep sequencing technology and bioinformatic approaches. As a result, 23 differentially expressed miRNAs in dry seeds were identified and confirmed between GM wheat and a non-GM acceptor. Notably, more differentially expressed tae-miRNAs between non-GM wheat varieties were found, indicating that the degree of variance between non-GM cultivars was considerably higher than that induced by the transgenic event. Most of the target genes of these differentially expressed miRNAs between GM wheat and a non-GM acceptor were associated with abiotic stress, in accordance with the product concept of GM wheat in improving drought and salt tolerance. Our data provided useful information and insights into the evaluation of miRNA expression in edible GM crops.

## Introduction

The global hectarage of genetically modified (GM) crops has increased 100-fold, from 1.7 million hectares in 1996 to 179.7 million hectares in 2015, for 17 to 18 million farmers in 28 countries. The latest data for 1996 to 2014 shows that GM crops contribute to food security, sustainability and climate change [1]. However, in many areas, cultivation of the GM crops was prevented because of the dominant ideological voices of the opponents, debates on their potential negative environmental impact, and their health risks to consumers. Although there is no sufficient scientific experimental evidence [2], the adverse effects of several GM crops that have been published in scientific journals still generate suspicion and controversy in the opinion of both the public and the scientific community [3–5]. Therefore, research assessing the potential risks associated with GM crops is essential for fast scientific adoption and general public acceptance.

The current evaluation system for GM crops is focused on proteins, fats, carbohydrates, toxins, and nutritional ingredients. However, miRNAs have not yet been taken into account in this system of evaluation. It was recently found that several gma-miRNAs are differentially expressed in GM soybean seeds compared with the non-GM control [6]. miRNAs have been widely demonstrated to play fundamental roles in gene regulation in most eukaryotes [7–10]. The first cross-kingdom regulation of gene expression between humans and edible plants by miRNAs was reported in 2012 [11]. Moreover, it was recently demonstrated that Brassica miRNAs can regulate the expression of human genes and proteins *in vitro* [12], which has raised new concerns regarding the miRNA components of GM crops.

miRNAs regulate gene expression by guiding target mRNA cleavage or by translational inhibition [7–10]. The targets of miRNAs include a large set of transcription factors (TFs) [13], such as Auxin Response Factors (ARFs) and QUAMOSA Promoter Binding Protein-Like (SPLs), which are involved in the regulation of miRNA expression in a negative feedback loop in *Arabidopsis* [14]. On the other hand, miRNAs are processed from hairpin precursors (pre-miRNAs), and pre-miRNAs are derived from primary transcripts (pri-miRNAs) transcribed from genomic DNA which is most likely regulated by TFs. An over-expressed transcription factor (*TaDREB3*) in barley affects the expression of miRNAs and other small non-coding RNAs in its leaf [15].

Due to their involvement in the regulation of many stress-related genes, many TFs were utilized in GM crops to improve crop tolerance to abiotic stresses. GM drought tolerance is an extremely important goal given that droughts will likely become more severe and more frequent as climate change impacts crop productivity, agriculture and society. So far, only 3 GM drought tolerant plants have been recommended for commercialization approval including GM maize in America, soybean in Argentina, and sugarcane in Indonesia. GM DroughtGard™ tolerant maize, first planted in the US in 2013, increased more than 15-fold from 50,000 hectares in 2013 to 275,000 hectares in 2014 and 810,000 hectares in 2015, reflecting a high farmer acceptance, at a 3-fold, year-to-year increase between 2014 and 2015 [1]. Wheat (*Triticum aestivum* L.) is one of the most important cereal crops worldwide, and it is used as a stable food grain and as a primary source of straw for animal feeding. Wheat production is severely affected by various abiotic stresses such as drought, salinity, low temperature, and heat, which results in an estimated 50–60% loss in grain yield annually [16]. Thus far, except for the glyphosate herbicide tolerance wheat (Mon71800), no other GM drought tolerant wheat has been approved for commercialization. Transgenic wheat transformed with a DREB TF (*GmDREB1*) from soybean (*Glycine max* L.), which is driven by an ubiquitin promoter showed strong tolerance to drought and salt [17, 18].

In this study, we investigated small RNAs from the dry seeds of a GM wheat line overexpressing *GmDREB1* and the non-transgenic control varieties using deep sequencing technology and bioinformatic approaches. Comparative analysis showed that 7 known tae-miRNAs were differentially expressed in the dry seeds between GM wheat and the non-GM acceptor. Combined with miRcheck prediction and experimental verification, 16 conserved and novel tae-miRNA candidates were also identified. This indicates that overexpression of the transcript factor *GmDREB1* affects the expression of miRNAs in wheat seeds. Most of the target genes of the differentially expressed miRNAs between GM wheat and the non-GM acceptor were associated with abiotic stress, in accordance with the product concept of the GM wheat line T349 [17, 18]. These results provided useful data for a bio-safety assessment of GM crops and valuable information for wheat miRNA research.

## 1. Materials and Methods

### 1.1 GM wheat samples and RNA isolation

The transgenic wheat line T349, non-transgenic acceptor Jimai 19 (J19) and comparable non-transgenic varieties Jimai 22 (J22) and Lumai 21 (L21) were provided by the Institute of Crop Science, Chinese Academy of Agricultural Sciences. The transgenic wheat line T349, transformed with *GmDREB1* from soybean, showed strong tolerance to drought and salt stresses [17, 18]. The transgenic and non-transgenic wheat varieties were planted in a field with water-saving measures in the Jinan province of China. The mature wheat seeds were harvested for RNA isolation. The main wheat cultivars, J22 and L21, were cultivated in the same climatic and geographic ecological region as J19. The total RNA was isolated from wheat seeds that were pooled from 3 individuals of the same transgenic or nontransgenic line using the Plant RNA Extraction kit (TaKaRa MineBEST) according to the manufacturer’s instructions. The quantity and quality of total RNA were evaluated using a NanoDrop 2000 Spectrophotometer and an Agilent 2100 RNA 6000 Nano kit.

### 1.2 Small RNA library construction and sequencing

The sRNAs were isolated from the total RNA using the TruSeq Small RNA Sample Preparation kit. The sRNA was ligated with a 3’-linker and electrophoresed on a 15% polyacrylamide gel. The 15–30 nt sRNAs were excised from the gel and recovered in 0.3 M NaCl. The recovered sRNAs were then ligated with a 5’-linker and subject to reverse transcription PCR. The RT-PCR products were separated on a 3.5% agarose gel. The 140–160 bp products were selected for library construction and submitted for sequencing on a Hi-Seq 2500 analyzer. The raw data from the small RNA libraries were deposited in the NCBI Sequence Read Archive (SRA) under accession No. SRP091415.

### 1.3 Bioinformatic analysis of high-throughput data

For deep sequencing data analysis, similar methods reported in a previous work were used in this section [6]. In brief, raw reads were cleaned by quality and adaptor trimming. Identical reads were collapsed and recorded for abundance. The SOAP programme was used for genome mapping. Only perfectly matched sRNAs with a length between 15–30 nt were subjected to further analysis [19]. The wheat (*Triticum aestivum* L.) genome was downloaded from the cerealsDB database (http://www.cerealsdb.uk.net/cerealgenomics/CerealsDB/copyright.php).

### 1.4 Differential expression analysis of known tae-miRNAs

Known wheat miRNA sequences were downloaded from miRBase (V21.0) [20]. The DESeq2 package [21] was used to determine the differentially expressed miRNAs, which were required to meet 3 prerequisites: raw reads abundance > 100, |log_2_ value| > 1 and FDR value < 0.001.

### 1.5 Novel miRNA prediction

Before novel miRNA prediction, small RNA reads were searched against the Rfam database (V11.0) to remove the known non-coding RNAs [22]. Customized Perl scripts, RNAfold and miRCheck programmes were used for context extraction, secondary structure determination and novel miRNA prediction [23]. Novel miRNA candidates were clustered with known miRNAs, including mature and precursor sequences, to find novel members in conserved families. Details of this process are described in a previous study [24].

### 1.6 Target prediction and function analysis

Target genes were predicted using psRNATarget [25], a plant sRNA target analysis server (http://plantgrn.noble.org/psRNATarget). Eight degradome libraries (GSM1606478, GSM1606479, GSM911923, GSM911924, SRR1197125, SRR1197126, SRR1197127, SRR1197128) were downloaded and analyzed for validating the cleavage sites in target genes using CleaveLand4 [26]. To further understand the function and classification of the predicted miRNA targets, GO classification of the target genes was conducted with WEGO web service (http://www.geneontology.org/), GO terms assigned to the query sequences and catalogued groups were produced based on their biological process, molecular functions, and cellular components.

### 1.7 RT-PCR assays

Differential expression of the tae-miRNAs was verified by qRT-PCR experiments. One microgram of total RNA was reverse-transcribed and amplified using miRcute miRNA First-Strand cDNA synthesis kit (Tiangen, KR201) and the miRcute miRNA qPCR detection kit (Tiangen, FP401). The comparative ΔΔCT method was used for the relative quantitation of these tae-miRNAs [27]. miR159 was selected as the endogenous reference gene for normalization [28]. For the validation of novel tae-miRNA candidates, a diluted cDNA template was used for RT-PCR assays with specific forward primers and a universal reverse primer. The amplified products were detected by electrophoresis on a 2% (w/v) agarose gel and validated by Sanger sequencing.

The expression profiles of some target genes of the differentially expressed miRNAs were analyzed by qRT-PCR analysis of a randomly selected set of genes. DNaseI-treated RNA was used for first-strand cDNA synthesis using Superscript III reverse transcriptase (Invitrogen, Carlsbad, CA, USA) and oligo (dT)18 primers according to the manufacturer’s protocol. The comparative ΔΔCT method was used for the relative quantitation of genes [27]. The Tubulin gene was amplified as an internal control. The primers used in this section are listed in **S1 Table**.

## 2. Results

### 2.1 Deep sequencing of small RNAs in wheat seeds

**Fig 1:**
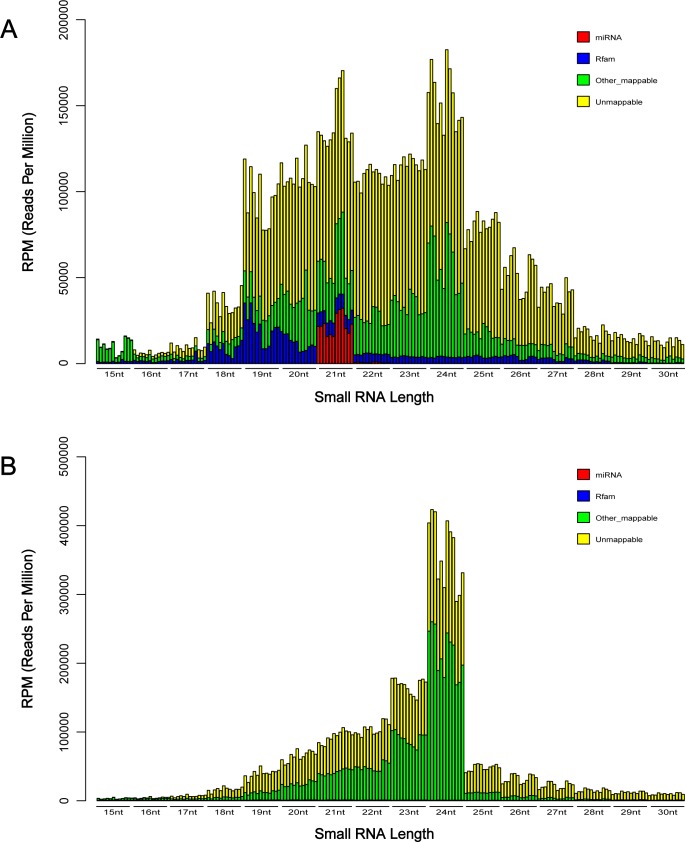
Length distribution of small RNA libraries. A: Length distribution of the total reads. B: Length distribution of the unique reads. For each class, 12 RPM values were arranged in order: T349_rep1, T349_rep2, T349_rep3, J19_rep1, J19_rep2, J19_rep3, J22_rep1, J22_rep2, J22_rep3, L21_rep1, L21_rep2, and L21_rep3.

Twelve small RNA libraries from T349, J19, J22 and L21 wheat cultivars, each with 3 replicates, generated 187,919,197 raw reads via the Illumina/Solexa deep sequencing platform. After the removal of low-quality reads and 3’ adaptor trimming, 159,723,105 (100%) clean reads, corresponding to 25,123,347 (100%) unique reads, were obtained for subsequent analysis. Genomic mapping results showed that 53,820,203 (33.7%) clean reads and 11,937,579 (47.5%) unique reads could find at least one perfectly matching locus in the wheat genome. We regarded 40,599,438 (25.4%) clean reads and 11,669,683 (46.4%) unique reads as unknown reads after searching against the miRBase and Rfam databases, which were used for novel miRNA prediction (**Table 1**). The length distribution of the clean reads showed that both 21-nt and 24-nt classes were dominant in the total reads (**Fig 1A**) and only the 24-nt class occupied the majority in the unique reads with obvious superiority (**Fig 1B**).

**Table 1:** Composition of small RNA libraries from wheat seeds of GM and non-GM plants.

		T349			J19			J22			L21		
		A	B	C	A	B	C	A	B	C	A	B	C
Raw reads	Total	19,203,574	14,806,199	17,440,475	17,302,501	17,735,423	17,113,284	10,471,928	11,234,870	10,844,006	16,663,383	18,475,545	16,628,009
High quality reads	Total	18,579,060	14,317,885	16,893,403	16,755,380	17,163,457	16,549,277	10,026,859	10,675,060	10,385,552	16,408,397	18,189,376	16,376,319
Clean reads(15–30 nt)	Unique	2,721,909	2,533,728	2,615,689	2,091,765	2,230,138	1,886,868	1,769,211	1,783,405	1,552,281	1,852,931	2,049,892	2,035,530
	Total	16,279,286	12,587,688	14,854,978	14,783,399	15,154,921	14,235,466	9,026,382	9,512,116	9,184,495	13,954,104	15,895,052	14,255,218
Not matching genome	Unique	1,340,349	1,237,502	1,301,946	1,149,935	1,214,177	1,069,609	921,192	954,777	837,046	993,653	1,108,576	1,057,006
	Total	10,006,079	7,872,719	9,104,624	10,217,273	10,499,254	9,987,590	5,653,864	5,993,448	5,670,558	9,816,304	11,266,819	9,814,370
Matching genome	Unique	1,381,560	1,296,226	1,313,743	941,830	1,015,961	817,259	848,019	828,628	715,235	859,278	941,316	978,524
	Total	6,273,207	4,714,969	5,750,354	4,566,126	4,655,667	4,247,876	3,372,518	3,518,668	3,513,937	4,137,800	4,628,233	4,440,848
Known tae-miRNAs	Unique	50	51	54	49	53	49	48	55	55	51	48	52
	Total	362,683	283,931	356,260	239,879	262,491	230,794	273,018	308,941	303,733	290,011	285,688	334,083
Matching Rfam database (exclude miRNAs)	Unique	23,704	21,117	22,795	25,326	25,151	24,991	17,178	18,022	16,883	23,418	25,084	24,227
	Total	1,673,743	1,112,800	1,534,831	1,316,541	1,232,379	1,247,230	479,033	511,618	551,157	1,083,226	1,301,795	1,176,412
Unknown reads	Unique	1,357,856	1,275,109	1,290,948	916,504	990,810	792,268	830,841	810,606	698,352	835,860	916,232	954,297
	Total	4,599,464	3,602,169	4,215,523	3,249,585	3,423,288	3,000,646	2,893,485	3,007,050	2,962,780	3,054,574	3,326,438	3,264,436

### 2.2 Expression profile of known tae-miRNAs in transgenic and non-transgenic wheat

**Fig 2:**
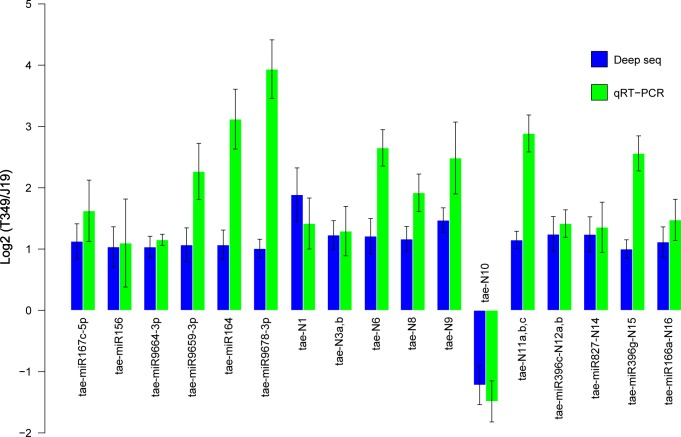
Verification of differentially expressed tae-miRNAs by qRT-PCR. The comparative ΔΔCT method was used for the qRT-PCR experiments and miR159 was selected as the reference.

To detect the known tae-miRNAs, the clean reads were aligned against the known tae-miRNAs represented in miRBase 21. As a result, 7 tae-miRNAs were up-regulated in the seeds of GM wheat T349 compared with the non-GM acceptor J19. Moreover, no down-regulated miRNAs were detected (**Table 2**). The log2 ratios varied from 1.01 to 1.20 and the maximum value was 1.20 for tae-miR319. Among these 7 known tae-miRNAs, 3 miRNAs, tae-miR167c-5p, tae-miR156 and tae-miR9661-3p were up-regulated only in GM wheat seeds compared to a non-GM acceptor, while there were no significant differences between the different non-GM wheat varieties tested in this study (L21/J19 and J22/J19). Four other tae-miRNAs were also differentially expressed between the non-transgenic wheat seeds. Compared with J19, tae-miR319 was up-regulated both in T349 and L21; tae-miR164 and tae-miR9678-3p were up-regulated both in T349 and J22 and tae-miR9659-3p was up-regulated in T349, L21 and J22 (**Table 2**). Meanwhile, the expressions of these known tae-miRNAs were confirmed by qRT-PCR (**Fig 2**). The differentially expressed miRNAs between the non-transgenic wheat varieties were not listed here, if they were not differentially expressed between T349 and J19.

**Table 2:** Differential expression of conserved tae-miRNAs in wheat seeds.

NO.	Mature miRNA	Sequence(5'-3')	Length (nt)	Log2 (T349/J19)		Log2 (L21/J19)		Log2 (J22/J19)		RPM in each bank											
										T349			J19			J22			L21		
1	tae-miR167c-5p	TGAAGCTGCCAGCATGATCTGC	22	1.13	↑	0.51	—	0.02	—	5.77	7.15	10.10	3.18	4.36	4.43	3.55	5.05	3.48	3.08	5.10	5.05
2	tae-miR156	TGACAGAAGAGAGTGAGCACA	21	1.04	↑	-0.36	—	0.51	—	3.99	2.94	3.77	2.30	2.05	1.62	3.77	2.00	2.72	1.15	0.82	1.61
3	tae-miR9664-3p	TTGCAGTCCTCGATGTCGTAG	21	1.03	↑	0.45	—	0.87	—	161.55	174.62	150.12	106.94	90.99	74.88	170.17	175.15	151.34	103.84	78.89	104.94
4	tae-miR319	TTGGACTGAAGGGAGCTCCCT	21	1.20	↑	1.02	↑	0.70	—	8.48	6.59	7.61	3.65	3.76	3.86	6.54	5.57	6.21	4.44	5.91	7.44
5	tae-miR9659-3p	TCCAATGGTTGTTCACGGCATC	22	1.07	↑	1.09	↑	1.23	↑	3.93	4.45	3.90	1.96	2.38	2.32	5.54	5.68	4.46	3.22	3.15	4.70
6	tae-miR164	TGGAGAAGCAGGGCACGTGCA	21	1.07	↑	0.81	—	1.62	↑	9.28	7.31	8.55	4.87	3.96	4.92	14.73	13.56	14.05	4.94	5.54	8.28
7	tae-miR9678-3p	TCTGGCGAGGGACATACACTGT	22	1.01	↑	0.17	—	1.42	↑	65.60	61.57	52.17	35.24	33.65	33.65	92.17	89.57	90.70	29.96	28.06	30.94

RPM, reads per million; ↑, up-regulated;—, no significant difference.

### 2.3 Predicted novel miRNAs in GM and non-GM wheat

**Fig 3:**
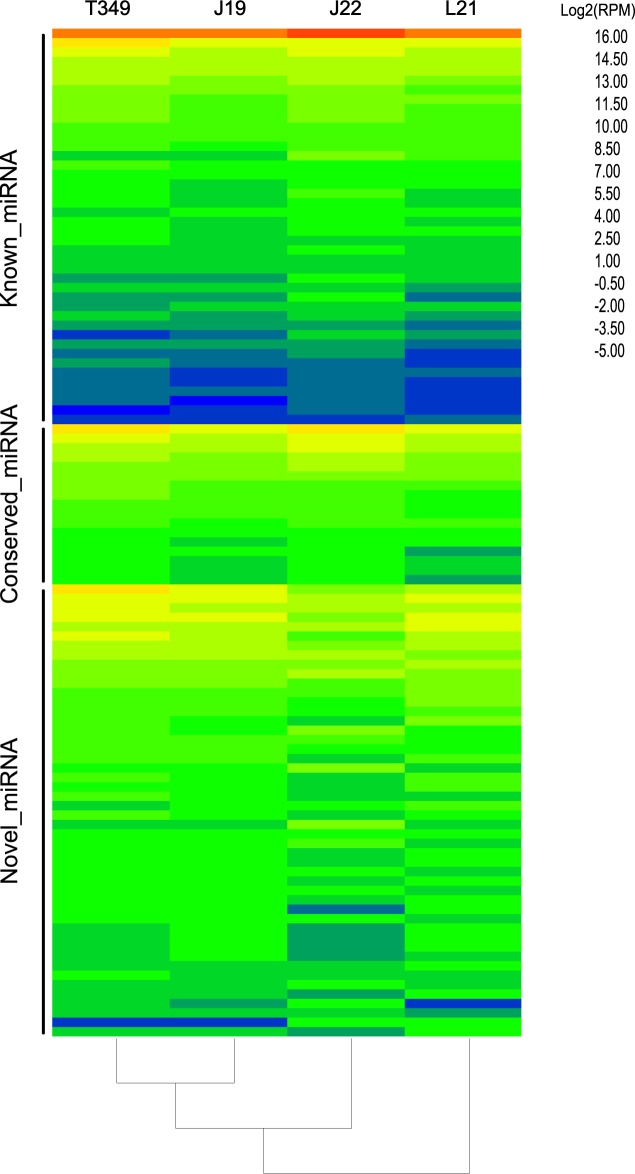
Heatmap of all miRNAs found in this study. miRNAs were classified into three categories: known, conserved and novel. For each panel, miRNAs were listed according to their abundance, which is Log2 (mean value of RPM).

In the 12 libraries, the sequenced miRNAs that were not reported in *Triticum aestivum* but were in *Aegilops tauschii*, *Brachypodium distachyon*, *Oryza sativan*, *Sorghum bicolor* or *Zea mays* according to miRBase 21, were defined as conserved miRNAs. The sequenced miRNAs that were not reported in *Triticum aestivum* or the other five monocotyledons referenced above were defined as novel miRNAs. Sixteen conserved miRNAs and 49 novel miRNAs were found in wheat seeds after sequence alignment **(Fig 3, S2 Table)**. All 49 candidate novel miRNAs and 16 conserved miRNAs possessed a perfect secondary stem loop structure (**S1 File, S2 File**). Because the miRNA* strand is degraded during typical miRNA biogenesis, we found both mature miRNA and miRNA* of the 8 miRNAs in 49 novel miRNAs and 6 conserved miRNAs (**S1 File**).

**Fig 4:**
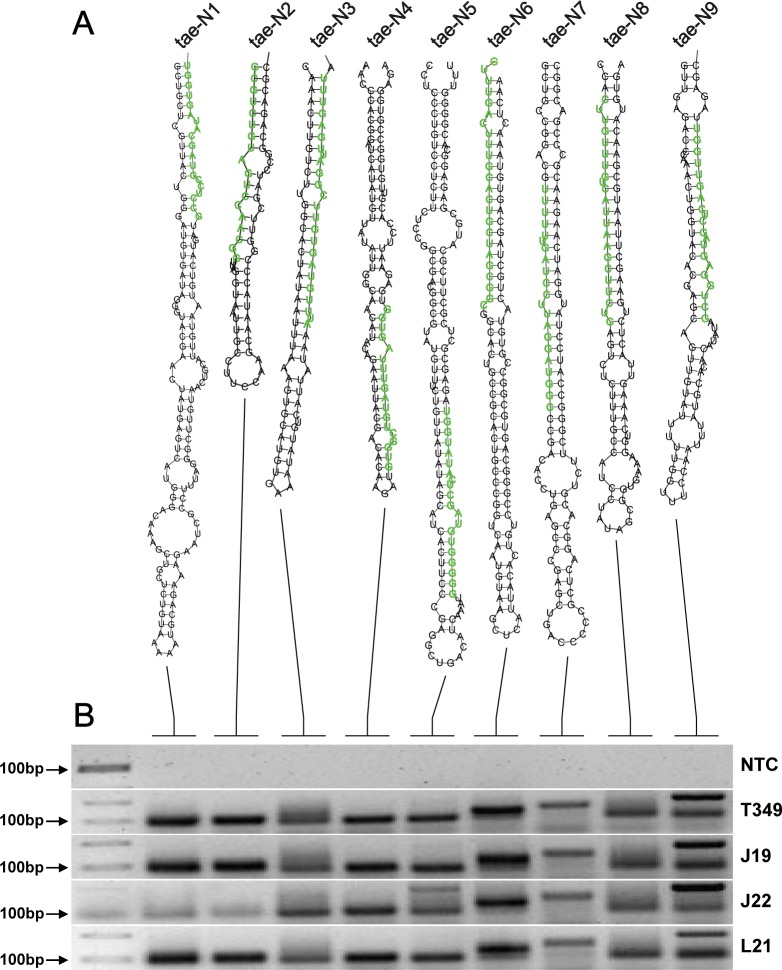
Novel tae-miRNA candidates found in this study. A: Hairpin structures of the novel tae-miRNA precursors predicted in GM wheat seeds. Mature miRNAs are indicated with green lowercase letters. B: Expression analysis of the novel tae-miRNA candidates by RT-PCR. NTC, no template control; T349, T349; J19, Jimai 19; L21, Lumai 21; J22, Jimai 22

Compared with the non-GM acceptor J19, 5 conserved miRNAs and 11 novel miRNAs were differentially expressed in the seeds of GM wheat T349 (**Table 3**). One novel miRNA was down-regulated and another 15 miRNAs were up-regulated in T349. In addition to T349, the conserved miRNAs ata-miR396c-5p, osa-miR164c and bdi-miR827-3p were also up-regulated in J22. The miRNAs ata-miR396c-5p and ata-miR166a-3p were also up-regulated in L21 and J22 compared with J19 (**Table 3**). Among the 11 novel miRNAs families, 2 miRNAs were differentially expressed only in GM wheat seeds, while no significant differences were observed between the different non-transgenic wheat varieties tested in this study (L21/J19 and J22/J19). We defined these two novel miRNAs as tae-N1 (5’GCCTCCGTAGCATAGTGGT3’) and tae-N2 (5’GCGTCTGTAGTCCAACGGT3’). The other 9 miRNA families were differentially expressed in both GM wheat T349 and the non-GM varieties J22 and/or L21 compared with J19. The expressions of some novel and conserved miRNAs were tested by qRT-PCR (**Fig 2**). The 11 novel miRNAs and the 5 conserved tae-miRNA candidates were subjected to experimental verification by RT-PCR and Sanger sequencing, and they were all positively identified in at least one library (**Fig 4, S3 File**).

**Table 3:** Differential expression of novel and conserved tae-miRNAs in wheat seeds.

NO.	Mature miRNA	Sequence(5'-3')	Length (nt)	Log_2_ (T349/J19)		Log_2_ (L21/J19)		Log_2_ (J22/J19)		RPM in each bank											
										T349			J19			J22			L21		
1	tae-N1	GCCTCCGTAGCATAGTGGT	19	1.89	↑	-0.12	—	-0.10	—	10.197	4.846	16.762	3.99	2.441	3.02	4.985	3.048	0.762	1.648	1.95	3.086
2	tae-N2	GCGTCTGTAGTCCAACGGT	19	1.32	↑	-0.01	—	-0.38	—	838.611	575.006	801.28	379.006	285.187	354.115	315.519	270.286	192.824	241.649	242.087	298.066
3	tae-N3a, b	ATTTGTAGTGTTCGGATTGAGTTT	24	1.23	↑	0.92	—	1.18	↑	10.872	14.935	12.319	7.102	6.73	4.706	15.731	14.718	11.65	8.456	7.36	11.364
4	tae-N4a, b, c, d, e, f, g	GTGGCTGTAGTTTAGTGG	18	1.29	↑	2.50	↑	-2.00	↓	11.302	22.164	9.895	14.881	9.237	15.665	9.306	9.566	6.423	6.736	5.095	4.7
5	tae-N5	GGGGGTGTAGCTCATATGGT	20	1.65	↑	-0.36	—	-1.80	↓	18.489	23.038	19.185	23.742	27.713	19.177	37.224	38.687	33.099	16.052	15.728	17.256
6	tae-N6	GTTTGACTTTGCACTGCTAGCGGC	24	1.21	↑	-2.27	↓	3.15	↑	2.088	1.668	3.433	6.29	5.74	7.656	0.221	1.471	1.85	10.534	9.499	10.031
7	tae-N7	TTTTTTGATCCTTAGGATGGC	21	1.15	↑	-0.06	—	1.26	↑	3.378	3.098	3.567	1.623	1.979	1.334	15.066	16.189	13.392	0.286	0.314	0.07
8	tae-N8	CTTGTTTGTCATTAAGCTTCTG	22	1.16	↑	-1.34	↓	-1.38	↓	6.204	6.673	7.135	7.778	12.141	9.202	4.431	5.992	3.919	4.944	6.291	5.471
9	tae-N9	GCTGGAGTAGCTCAGTTGGT	20	1.47	↑	-0.13	—	-2.13	↓	14.435	13.902	10.77	6.29	6.928	7.024	15.731	16.189	16.549	4.156	5.284	5.471
10	tae-N10	GCGGGAGGCACGGGGTTCG	19	-1.22	↓	0.97	—	-2.37	↓	24.878	34.16	27.061	14.949	16.298	12.714	7.311	4.625	4.681	4.228	4.718	4.279
11	tae-N11a, b, c	ATCAGAGTGGCGCAGCGGA	19	1.15	↑	0.02	—	-2.36	↓	26.291	24.865	27.802	11.972	9.831	10.818	3.434	1.366	2.395	7.381	8.052	7.506
12	tae-miR396c-N12a, b	TCCACAGCTTTCTTGAACTG	20	1.24	↑	1.03	↑	1.25	↑	6.941	10.565	5.789	3.652	2.837	4.636	9.084	9.777	7.839	4.371	5.85	7.576
13	tae-miR164c-N13	TGGAGAAGCAGGGCACGTGCT	21	1.38	↑	0.32	—	1.62	↑	7.739	6.355	8.078	3.314	2.969	3.442	9.084	9.251	11.541	2.938	3.271	3.156
14	tae-miR827-N14	TTAGATGACCATCAGCAAACA	21	1.24	↑	0.00	—	1.39	↑	6.941	7.547	7.404	4.193	2.969	3.371	9.416	5.992	12.085	3.368	2.327	2.455
15	tae-miR396g-N15	TCCACAGGCTTTCTTGAACTG	21	1.00	↑	0.54	—	1.13	↑	101.97	101.845	90.676	58.308	56.681	54.23	129.62	131.936	108.552	56.9	60.396	72.254
16	tae-miR166a-N16	TCGGACCAGGCTTCATTCC	19	1.12	↑	1.38	↑	1.20	↑	48.527	57.357	46.314	29.627	35.236	30.416	88.739	87.046	63.585	39.056	36.363	54.786

↑, up-regulated; ↓, down-regulated;—, no significant difference. No.1-11, novel miRNAs; No. 12–16, conserved miRNAs.

### 2.4 Target genes of up- and down-regulated miRNAs in GM and non-GM wheat

miRNAs regulate target genes involved in plant development and stress response. miR167 target auxin response factor mediated plant root development [29, 30]. miR156 targets SQUAMOSA promoter binding protein-like (SPL) TFs related to flowering time, phase change and leaf initiation rate [31, 32]. miR319 targets TCP transcription factors and miR164 targets NAC domain gene, which contributes to leaf development [33]. Moreover, these target genes are all involved in stress response [34–38] **(Table 4)**. To reveal the function of the predicted novel miRNAs and the conserved miRNAs, we predicted their targets using psRNATarget (http://plantgrn.noble.org/psRNATarget/). As is common for wheat, some predicted targets were functionally unknown and most miRNAs were found to target more than one gene **(S3 Table).** Part of these cleave sites were validated by wheat degradome data (**S4 Table**). T-plots of the identified targets were shown in **S1 Fig**. The expression profiles of some target genes of differentially expressed miRNAs were analyzed by qRT-PCR, and some target genes were down-regulated in T349 compared with J19 (**S2 Fig**). GO analysis results of all the target genes of the differentially expressed miRNAs in GM and non-GM wheat showed that many targets were related to water transport and responses to oxygen-containing compounds **(S3 Fig)**, suggesting that the corresponding miRNAs may be involved in drought/salt stress and oxidative stress.

**Table 4:** Target prediction of known tae-miRNAs in wheat seeds.

miRNA	Target accession				Known annotation	New annotation
tae-miR167c-5p	TC402576	CV773862	TC422705		Auxin Response Factor	No results.
tae-miR156					Squamosa Promoter-binding-Like protein (SPL)	Teosinte glume architecture 1,
	TC453361	CK196549	AL810223	TC409846		Glycosyl/glycerophosphate transferase,
	DR739383	TC384445	TC420438	TC412204		Predicted permease,
	TC441570	TC373290	TC398965	TC460639		Cob(I)alamin adenolsyltransferas,
	TC372857	TC390294	CA741955	TC370322		Cytochrome P450,
	TC413555	CA612886				Telomere binding protein,
						Initiator binding protein.
tae-miR9664-3p					Not available	NB-ARC domain containing protein,
	CN008390	CN007906	CJ899791	DR734475		Nodulin-like protein,
	CK202829	CK203152	BE425456	BE604149		NB-ARC domain containing protein,
	CD876018	CA499346	CV779224	TC436817		NBS-LRR resistance-like protein,
	CK193467	TC451016	BJ245571	BE585521		Resistance protein RGA1R,
	CV772438	TC401974	DR738720	TC379417		LRR19,
						Alternative splicing regulator.
tae-miR319					TCP and MYB transcription factor	Glycosyl transferase,
	TC407332	CK212140	TC421314	TC368630		Histone H2B,
	BF485310	CA484819	TC455115	BE517710		Acyl-CoA synthetase,
	GH728978	TC438746	CA630893	TC398226		Trans-cinnamate 4-monooxygenase,
	TC432120	CK215833	CJ863381			Type 1 non specific lipid transfer protein precursor,
						Transcription factor PCF8,
						Ribosomal protein L3-A2-II.
tae-miR9659-3p	TC438538	CA606192	CV779210	TC382784	Not available	Anaeromyxobacter dehalogenans,
	TC454472	TC371921	TC438010	BQ904504		Glutathione transferase,
	BE401763	CA639001	TC393061	TC371776		HvPIP1;5 protein,
	TC370293	CD875198	TC383120			Aquaporin PIP1-2,
						Inositol-1,4,5-triphosphate-5-phosphatase.
tae-miR164	TC416811	CA642340	TC390810	TC376198	NAC domain-containing protein	
	TC394945	TC373635	TC371535	TC410195		
	TC429623	CA704421	TC393137	TC421735		Efflux ABC transporter permease protein,
	TC369110	CA681504	TC398164	TC406273		Harpin-induced protein 1 containing protein,
	TC394481	TC371551	TC370694	TC382290		Mitogen-activated protein kinase homolog 1,
	TC375350	DR741669	TC368951	DR741517		ZmRR2 protein,
	TC378810	BE515854	TC435250	TC430604		Phytosulfokine-alpha 1 precursor,
	CK211831	TC445043	TC458082	CA648036		OTU-like cysteine protease family protein,
	TC408311	TC407532	TC430881	TC407133		Sugar transport protein.
	TC404361	TC411029	TC384650	CK198447		
	CK216067	BJ266172				
tae-miR9678-3p	TC427317	DR736808	TC381152	CJ648199	Not available	F-box protein-like.

## 3. Discussion

### 3.1 Over-expressed transcription factor (*GmDREB1*) in wheat affects the expression of miRNA in seeds

The global area for the cultivation of transgenic plants has grown continuously during the past two decades, despite constant controversy concerning their unforeseeable biosafety issues [1]. The application of transgenic technology has led to significant changes in modern agriculture and this trend seems irreversible [1]. Because of the dynamic property of RNA molecules, it is reasonable to propose that some endogenous RNAs might be diverse in transgenic plants. To date, very few reports have discussed the RNA ingredient content difference between GM and non-GM plants [6, 15]. Herein, we investigated the differential expression of miRNAs in wheat seeds from a GM line (T349), its non-GM receptor (J19) and local major wheat cultivars (J22 and L21) using high throughput sequencing technology. As a result, *GmDREB1*over-expression in wheat affects the expression of miRNAs in seeds. Other reports also mentioned the relationship between miRNAs and DREB. The expression levels of *DREB1A* and *DREB2A* were relatively induced in miR408 overexpressed plants compared to the vector control upon drought stress [39]. An over-expressed transcription factor (*TaDREB3*) in barley affects the expression of miRNAs and other small non-coding RNAs in its leaf [15].

In this study, the degree of variance, including miRNAs (**Fig 2**) and sRNA variance (**S4 Fig**), between cultivars was much higher than that induced by the transgenic event. Twenty-three differentially expressed miRNAs were found and confirmed between the T349 and J19 lines. Moreover, some of the miRNAs affected by DNA manipulation overlapped with the differentially expressed miRNAs related to varietal influence. Further analysis showed that most target genes of these differentially expressed miRNAs were associated with abiotic stress. This is understandable because a *GmDREB1* transcription factor was introduced in the transgenic line T349 to elevate its drought and salt tolerance ability [17, 18].

### 3.2 Target genes of the differentially expressed miRNAs were associated with abiotic stress

Differentially expressed miRNAs were observed between the GM wheat T349 and the non-GM acceptor J19. Several known miRNAs (miR319, miR164, miR167, and miR156) are involved in drought response. miR319 regulates TCP transcription factors [33] and the NAC domain gene, which contributes to leaf development and drought stress response [38]. Moreover, the NAC domain gene is known as the target gene of miR164 in rice [40], and TCP genes have been shown to regulate miR164 in *Arabidopsis* [34]. It was reported that miR319 also target MYB transcription factors [41] that play significant roles in stress responses. miR167 regulates its target gene, ARF, which mediates plant root development [30, 37] and responds to drought stress [35]. Another identified target of miR167, IAA-Ala Resistant3 (IAR3) has the same roles as ARF in drought stress and root development [29]. miR156 targets SQUAMOSA promoter binding protein-like (SPL) TFs, which control flowering time, phase change and leaf initiation rate [31, 32, 42]. The miR156-SPL9-DFR pathway coordinates the relationship between development and abiotic stress tolerance in plants. Blocking the miR156 signalling pathway with 35S::MIM156 (via target mimicry) increased the sensitivity of the plant to stress treatment, whereas the overexpression of miR156 increased stress tolerance [36]. In transgenic barley overexpressing the drought tolerant gene *TaDREB3*, miR156 was over two-fold up-regulated when comparing with the non-transgenic control. Thus these known miRNAs up-regulated in GM wheat may enhance the stress tolerance of transgenic wheat. In another study, miRNA microarray analysis showed that miR156, miR167, miR164, miR319, miR396 and miR166 were up-regulated in leaf or root of bread wheat under drought stress [43]. GO analysis of all the target genes of differentially expressed miRNAs in the GM and non-GM wheat lines show that many targets are related to water transport and response to oxygen-containing compounds (**S3 Fig**), suggesting that the corresponding miRNAs may be involved in drought/salt stress and oxidative stress.

### 3.3 Possible environmental risks of differentially expressed miRNAs in GM wheat

A controversial and attention-drawing issue is whether the exogenous miRNAs of GM crops could regulate human gene expression by cross-kingdom RNAi, which has been observed in both animal and plant systems. In animal systems, there are a few instances of cross-kingdom gene silencing. One example is that RNAi can be induced by soaking *Caenorhabditis elegans* worms in RNA solutions or by feeding them antisense RNA-expressing bacteria, such as *Escherichia coli* [44]. Plants transfer RNAi signals into interacting organisms, such as filamentous fungi, oomycetes, nematodes, parasitic plants, and pests [45–48] to silence their genes; however, it is not confirmed whether miRNA mediates human-plant interactions. Although it was reported that exogenous rice MIR168a is present in the human and mouse sera and the exogenous MIR168a inhibits human/mouse *LDLRAP1* gene expression in liver [11], many subsequent reports have also questioned this controversial result [49–52]. It was recently demonstrated that Brassica miRNAs could regulate the expression of human genes and proteins, but only *in vitro* [12].

In this study, the targets of 23 differentially expressed miRNAs in GM wheat seeds were predicted in human (**S5 Table**) and chicken (**S6 Table**), both of which may eat GM wheat seeds. The top 50 predicted targets with the highest scores of 23 differentially expressed miRNAs in GM wheat seeds were mainly involved in the regulation of metabolic process, including primary, macromolecule and RNA metabolic processes in humans (**S5A Fig**) Moreover, they were primarily involved in epithelial cell differentiation in chicken (**S5B Fig**). However, our study did not conclude whether there are possible environmental risks of eating GM wheat seeds exist until it was the cross-kingdom RNAi was proven to be induced between animal/human-plant *in vivo*, and the differentially expressed miRNAs target these genes as predicted.

In addition to miRNA, sRNA includes other classes, such as small interfering RNAs, a second class of small regulatory RNAs that can direct DNA methylation and regulate salt tolerance and disease resistance [53, 54]. Moreover, long noncoding RNAs that emerged as important regulators of many biological processes in animals were identified in higher plants [55]. In this study, besides the 23 miRNAs discussed above, other sRNAs may be taken into account during GM crop evaluations in the future. The function of these different types of sRNA will gradually be identified in future studies. Moreover, the targets of some miRNAs were not predicted. For example, tae-N1 and tae-N2 from this study may function by interacting with other mRNA or lincRNA. The complications of sRNA justify taking sRNA into account in the evaluation system for GM crops. Our findings provide useful data for assessing the potential risks associated with GM crops and provide valuable information for wheat miRNA research.

## Supporting information

S1 Fig T-plots of cleavage sites in target genes verified by degradome data. (PDF)

S2 Fig Relative expression profiling of some targets of miRNAs in T349 compared with J19. (PDF)

S3 Fig GO analysis of target genes of conserved and novel tae-miRNAs in wheat seeds. (PDF)

S4 Fig Volcano plot demonstrating the differential expression of tae-sRNAs in GM wheat seeds. (PDF)

S5 Fig GO analysis of top 50 targets of 23 differentially expressed miRNAs in human and chicken. (PDF)

S1 File Alignments between mature miRNAs and their precursors. (TXT)

S2 File Secondary structure of 49 novel miRNAs and 6 conserved miRNAs. (PDF)

S3 File Sanger sequencing results of novel miRNAs. (DOCX)

S1 Table Primers used for qRT-PCR experiments in this study. (XLSX)

S2 Table The sequences of the conserved and novel miRNAs found in this study. (XLSX)

S3 Table Targets of the conserved and novel tae-miRNAs predicted in wheat seeds. (XLSX)

S4 Table Verification of cleave sites in target genes by degradome data. (XLSX)

S5 Table Targets of 23 differentially expressed miRNAs predicted in human (XLSX)

S6 Table Targets of 23 differentially expressed miRNAs predicted in chicken. (XLSX)
