# Supplementary material for: GmDREB1 overexpression affects the expression of microRNAs in GM wheat seeds
Source: PLoS One. 2017 May 1;12(5):e0175924. doi: 10.1371/journal.pone.0175924 (PMC5411081; doi:10.1371/journal.pone.0175924)

```
5' AGCAAGUGCCCUGCUUCUCCA 3' Transcript: TC410195:739-759 Slice Site:750
    ||| ||||||||||||||||
3' ACGUGCACGGGACGAAGAGGU 5' Query: tae-miR164

Degradome data file: SRR1197127
Degardome Category:  0
Degradome p-value:   0.00141301193607313
```

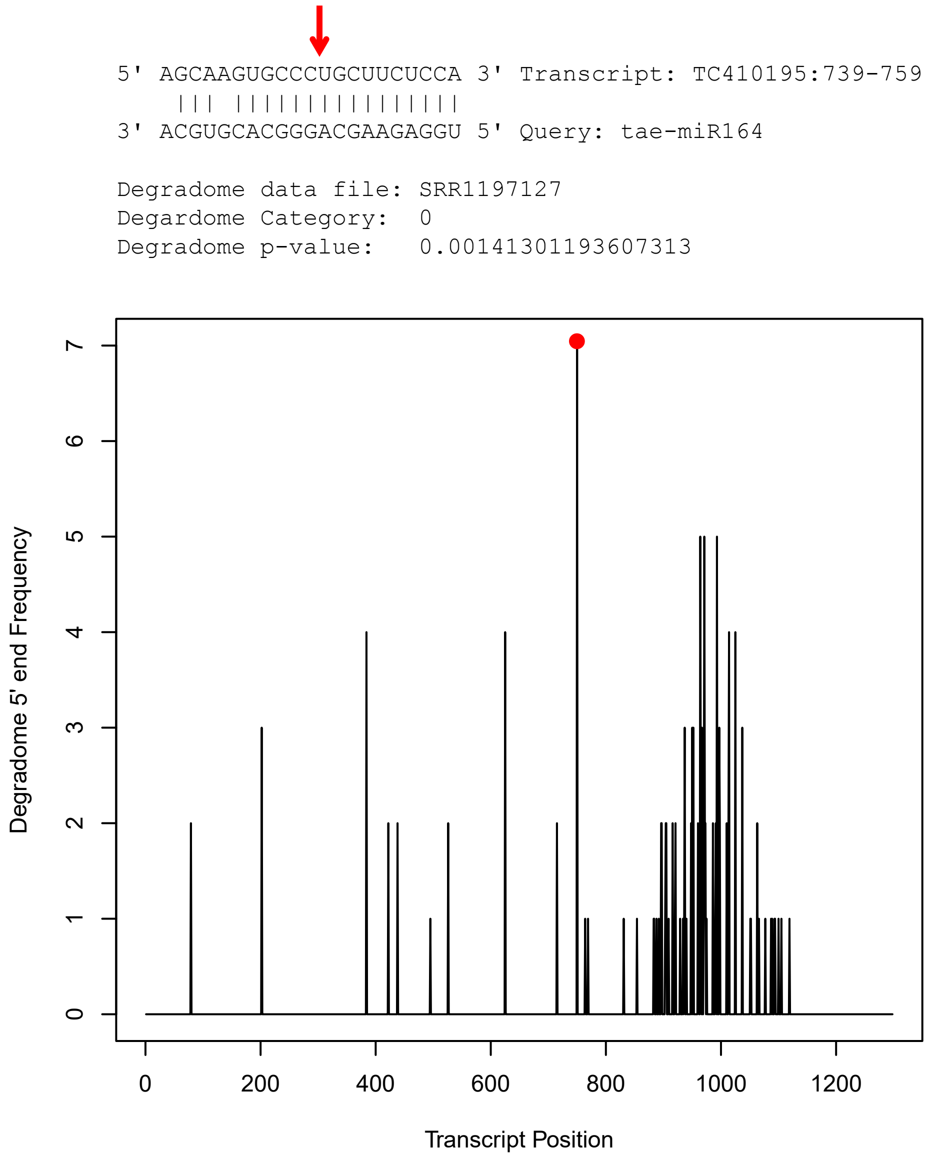

```
5' AGCAAGUGCCCUGCUUCUCCA 3' Transcript: TC416811:106-126 Slice Site:117
    ||| ||||||||||||||||
3' ACGUGCACGGGACGAAGAGGU 5' Query: tae-miR164

Degradome data file: SRR1197126
Degardome Category:  0
Degradome p-value:   0.000419649287547452
```

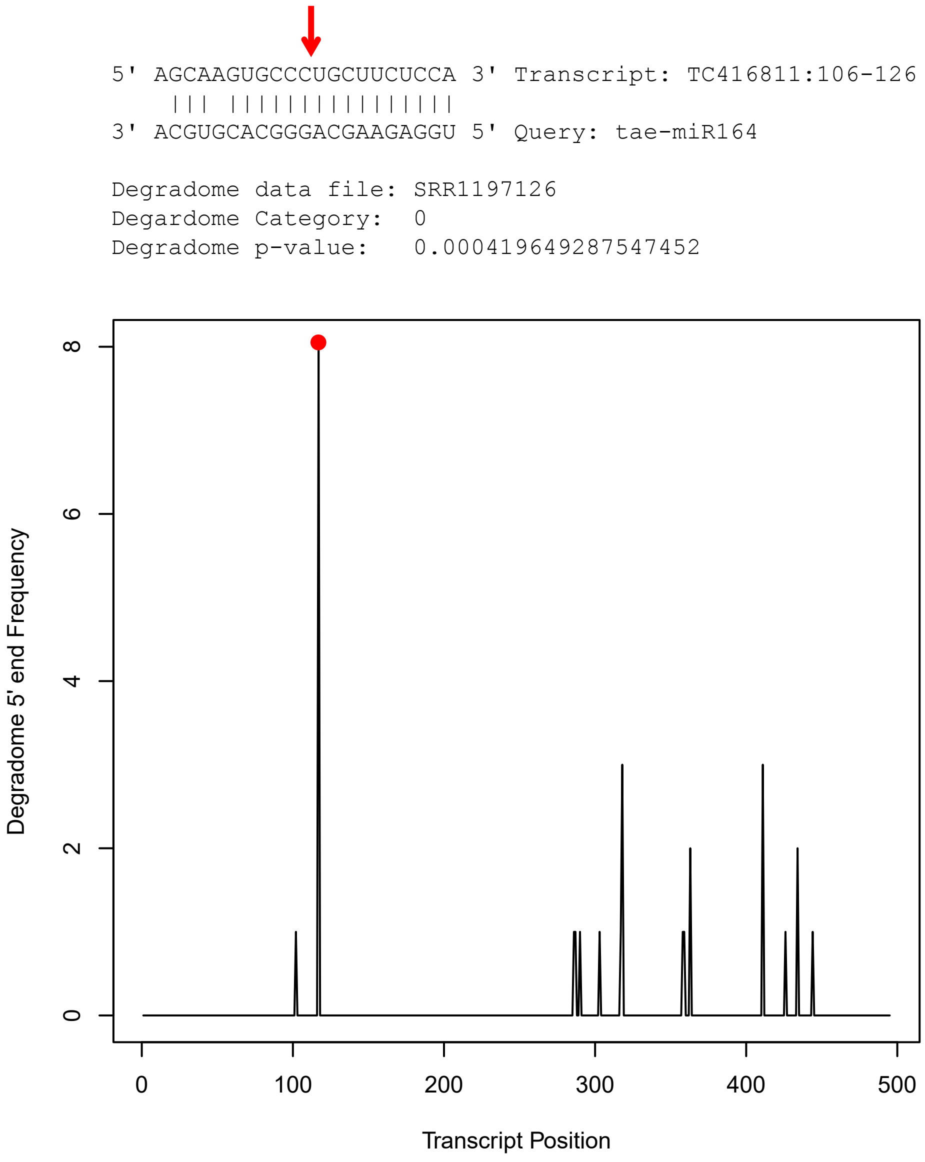

```
5' AGCAAGUGCCCUGCUUCUCCA 3' Transcript: TC390810:340-360 Slice Site:351
   |||| ||||||||||||||||
3' UCGUGCACGGGACGAAGAGGU 5' Query: tae-miR164c-N13
```

Degradome data file: SRR1197128
Degardome Category: 0
Degradome p-value: 0.000841342606561657

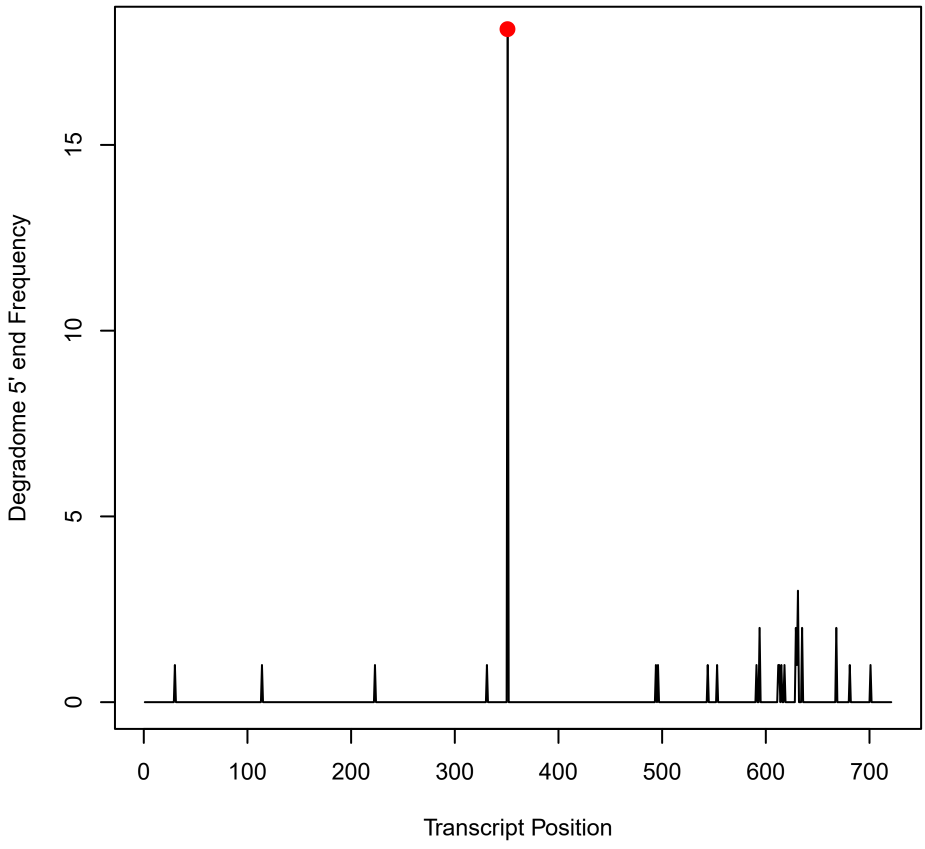

```
5' AGCAAGUGCCCUGCUUCUCCA 3' Transcript: TC394945:240-260 Slice Site:251
   |||| ||||||||||||||||
3' UCGUGCACGGGACGAAGAGGU 5' Query: tae-miR164c-N13

Degradome data file: SRR1197126
Degardome Category:  0
Degradome p-value:   0.00293384938232144
```

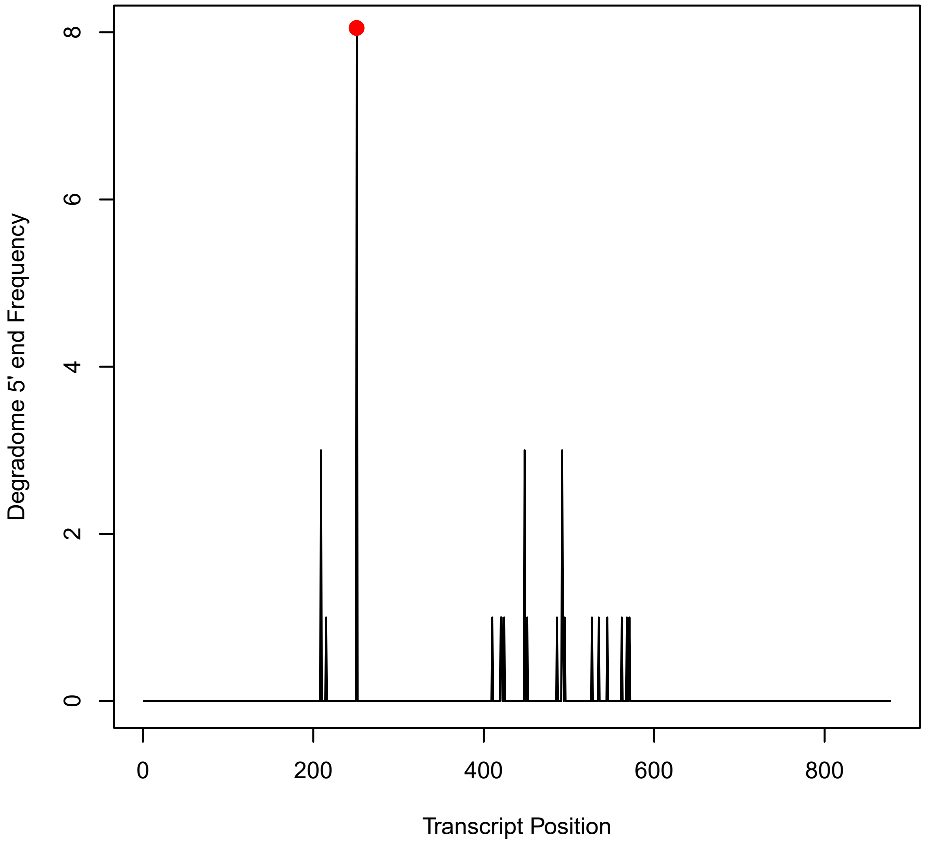

```
5' AGCAAGUGCCCUGCUUCUCCA 3' Transcript: TC410195:739-759 Slice Site:750
   |||| ||||||||||||||||
3' UCGUGCACGGGACGAAGAGGU 5' Query: tae-miR164c-N13
```

Degradome data file: SRR1197126
Degardome Category: 0
Degradome p-value: 0.00167754081262195

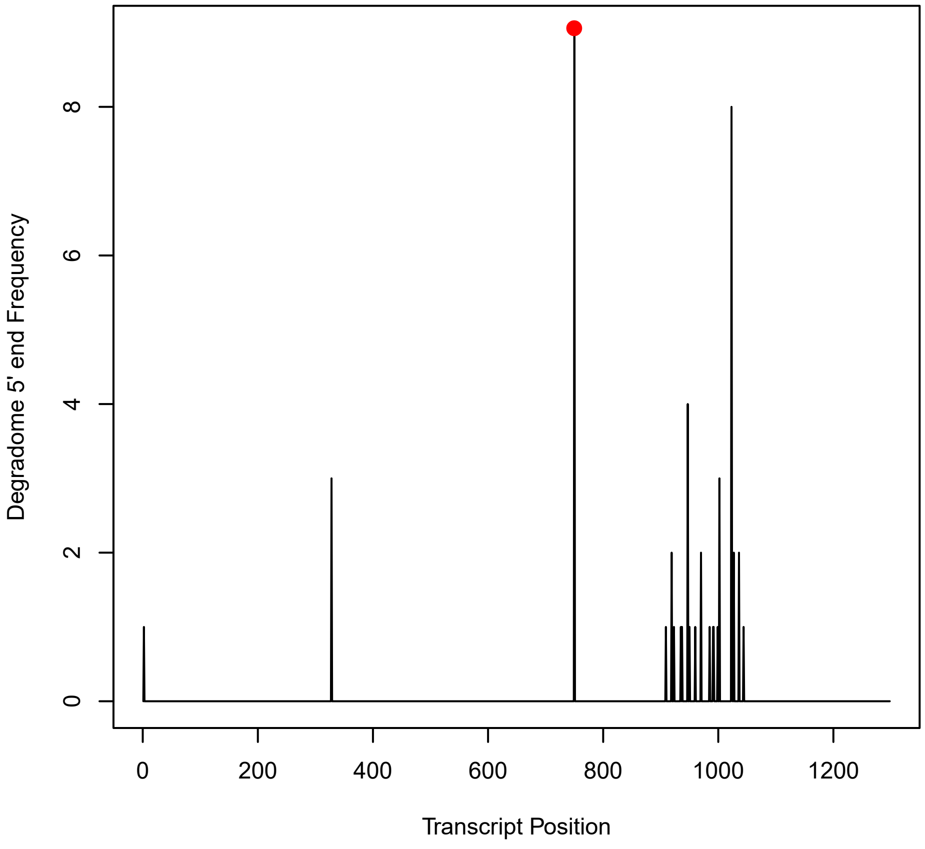

```
5' AGCAAGUGCCCUGCUUCUCCA 3' Transcript: TC429623:744-764 Slice Site:755
   |||| ||||||||||||||||
3' UCGUGCACGGGACGAAGAGGU 5' Query: tae-miR164c-N13

Degradome data file: SRR1197126
Degardome Category:  0
Degradome p-value:   0.00251525562000277
```

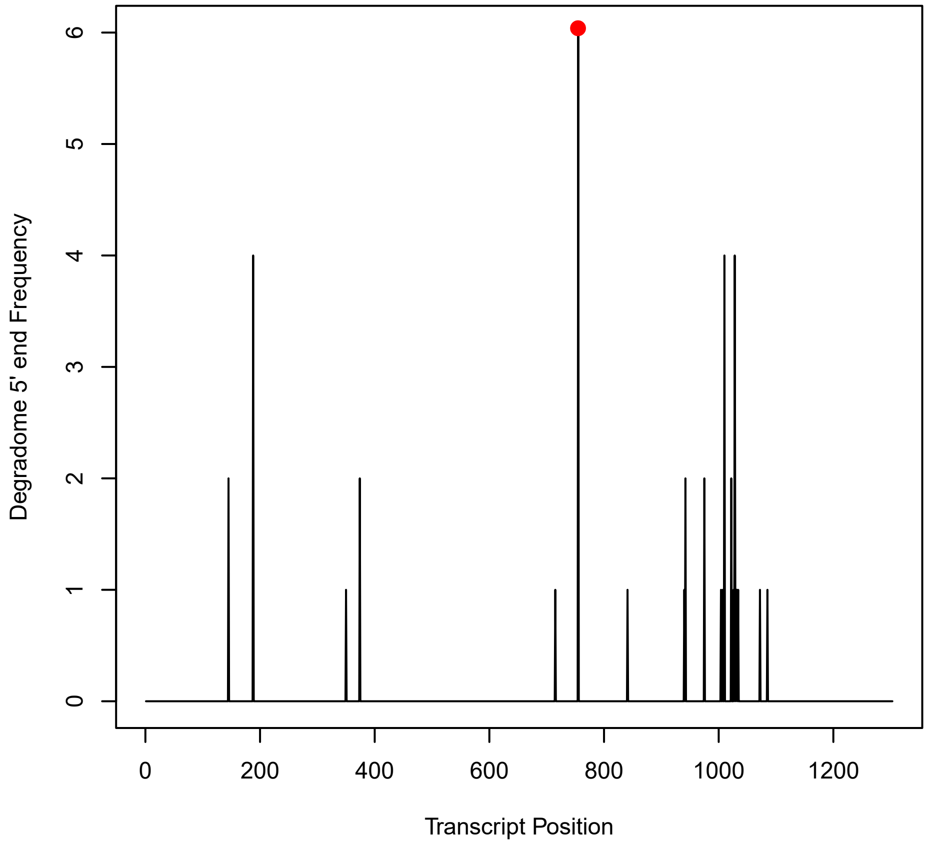

```
5' UAGUUCAAGACAAGCUGUGGA 3' Transcript: CA603835:311-331 Slice Site:322
   o|||||||||  ||||||||||
3' GUCAAGUUCU-UUCGACACCU 5' Query: tae-miR396c-N12a,b

Degradome data file: GSM911924
Degardome Category:  0
Degradome p-value:   0.00111846811950223
```

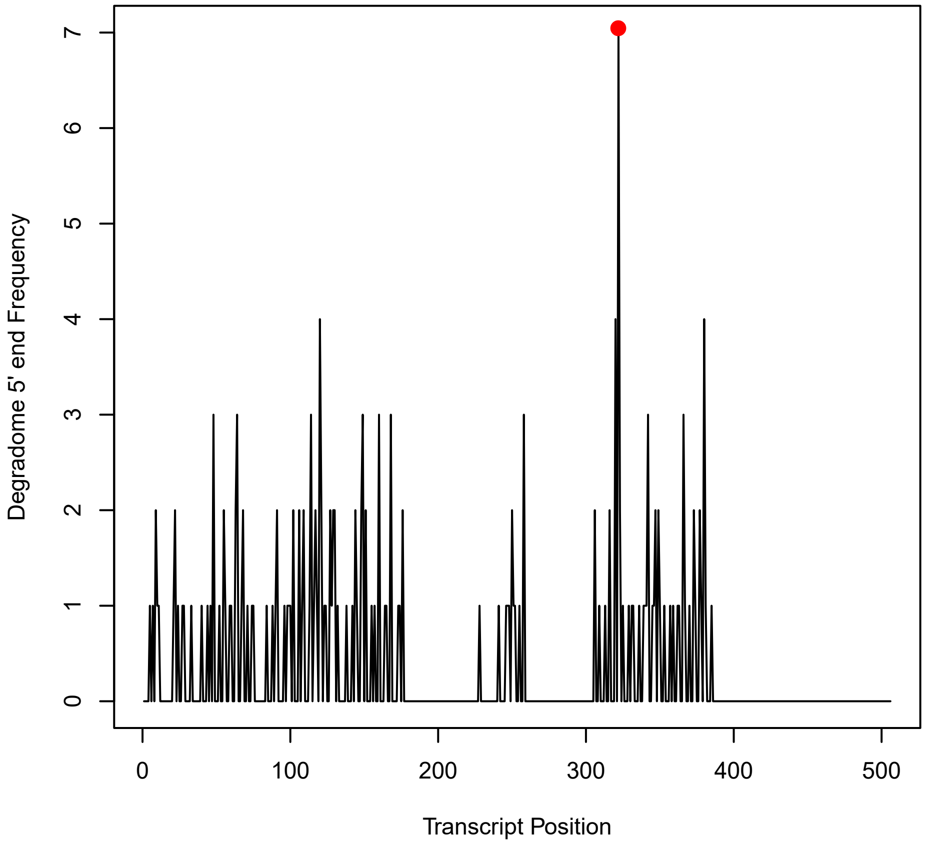

```
5' UGGUUCAAGACAAGCUGUGGA 3' Transcript: TC456289:1142-1162 Slice Site:1153
    o|||||||| ||||||||||
3' GUCAAGUUCU-UUCGACACCU 5' Query: tae-miR396c-N12a,b

Degradome data file: GSM911924
Degardome Category:  0
Degradome p-value:   0.00335165284487493
```

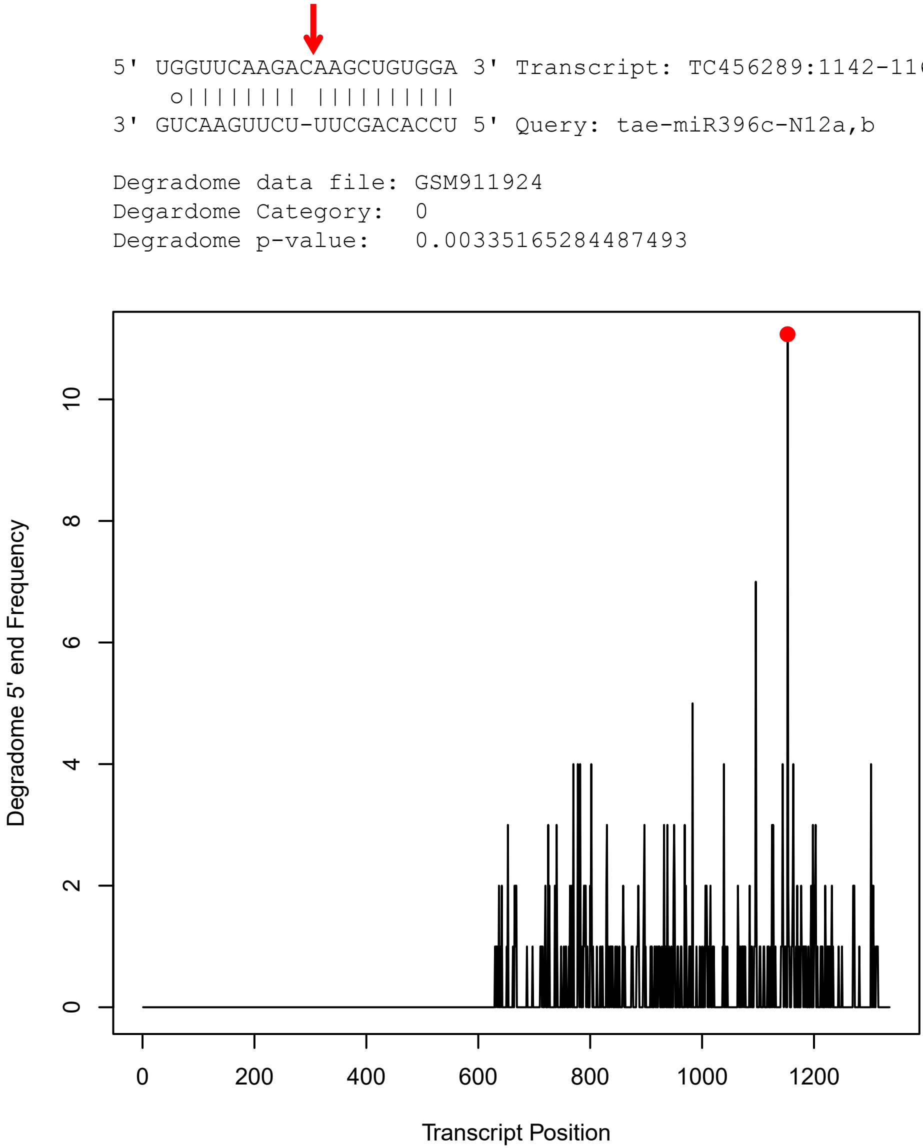

```
5' CCGUUCAAGAAAGCCUGUGGA 3' Transcript: CJ645826:90-110 Slice Site:101
   | |||||||||||||||||||
3' GUCAAGUUCUUUCGGACACCU 5' Query: tae-miR396g-N15

Degradome data file: GSM911924
Degardome Category:  0
Degradome p-value:   0.000559390518627301
```

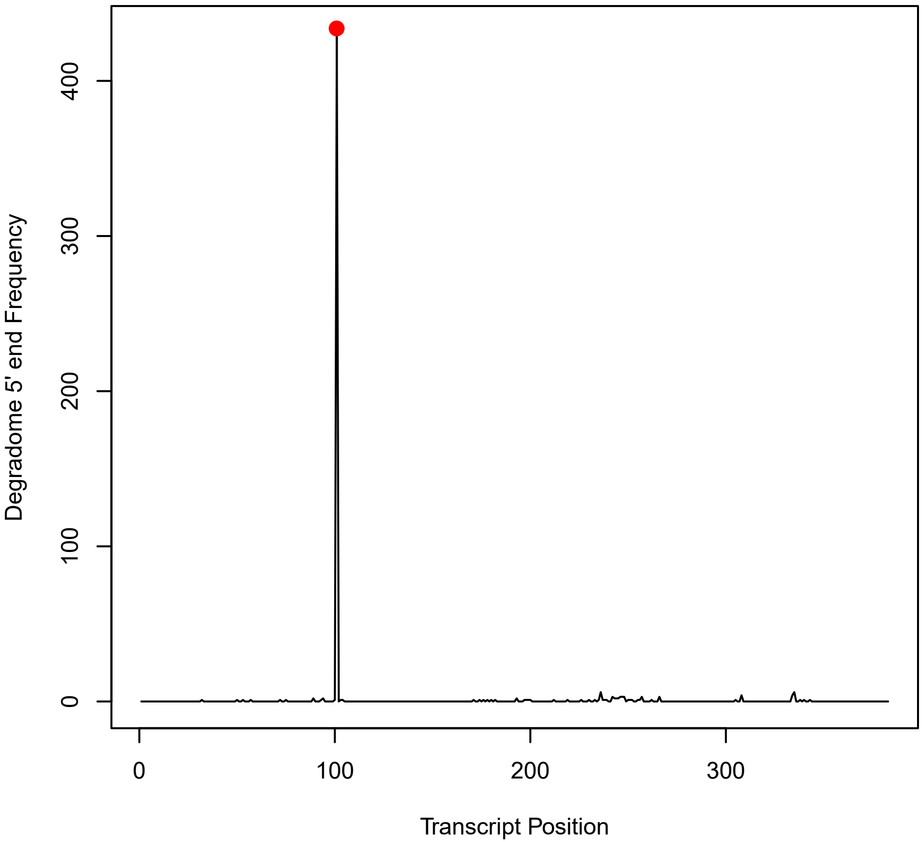

```
5' CCGAUCAAGAGAGCCUGUGGA 3' Transcript: CK209519:536-556 Slice Site:547
       ||||||o||||||||||
3' GUCAAGUUCUUUCGGACACCU 5' Query: tae-miR396g-N15

Degradome data file: GSM911924
Degardome Category:  0
Degradome p-value:   0.00223568526807016
```

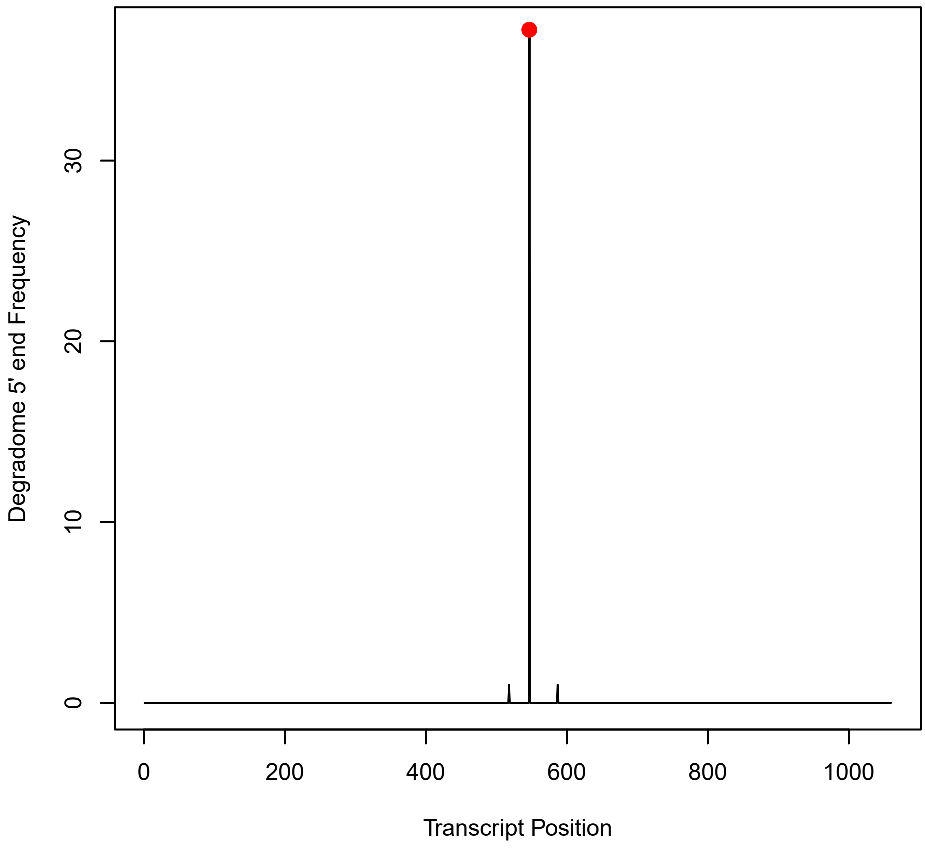

```
5' UCGUUCAAGAAAGCCUGUGGA 3' Transcript: TC421041:242-262 Slice Site:253
     |||||||||||||||||||
3' GUCAAGUUCUUUCGGACACCU 5' Query: tae-miR396g-N15

Degradome data file: GSM911923
Degardome Category:  0
Degradome p-value:   0.00104807042237054
```

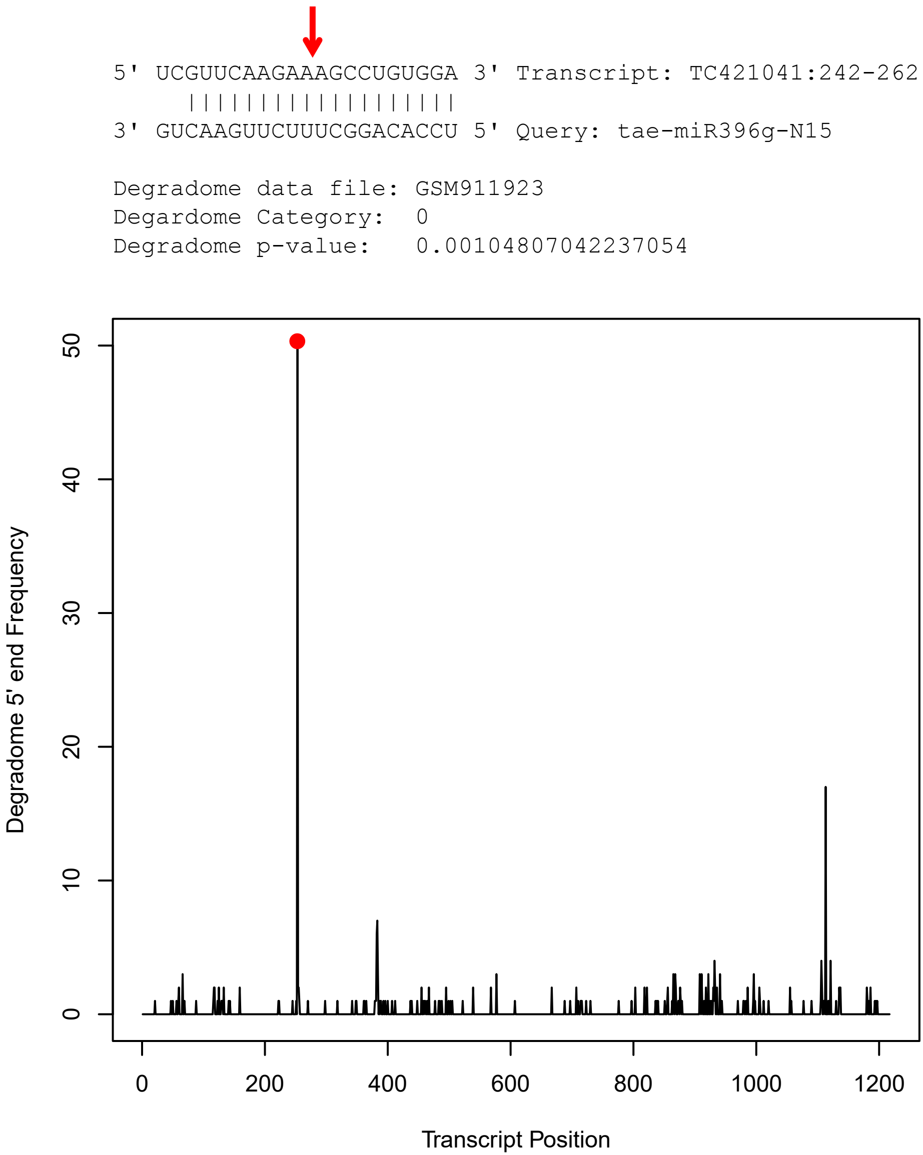

Supplement: S1 Fig — (PDF) [file pone.0175924.s001.pdf]
