# Supplementary figures and images for: GmDREB1 overexpression affects the expression of microRNAs in GM wheat seeds
Source: PLoS One. 2017 May 1;12(5):e0175924. doi: 10.1371/journal.pone.0175924 (PMC5411081; doi:10.1371/journal.pone.0175924)

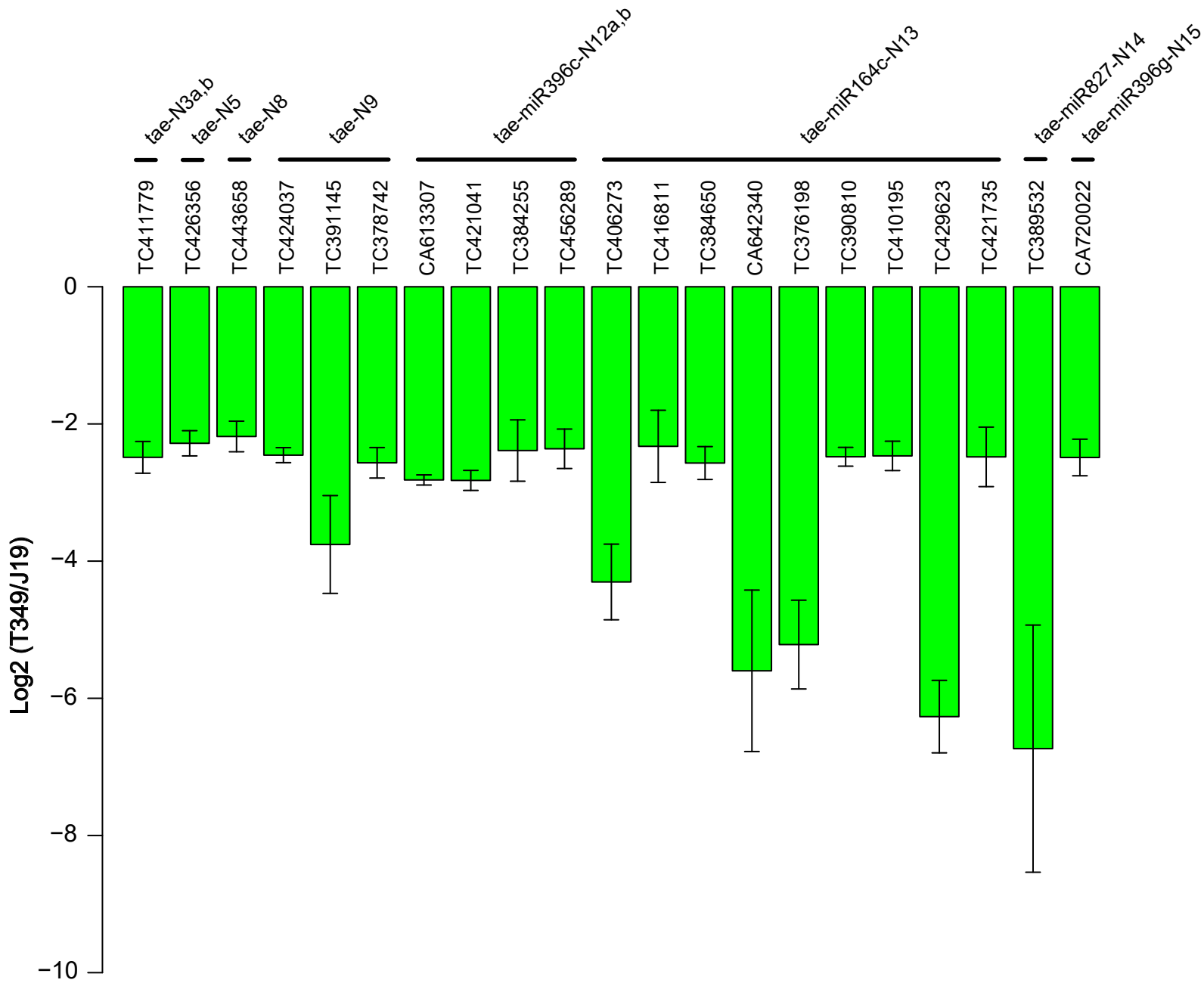

Supplement: S2 Fig — (PDF) [file pone.0175924.s002.pdf]

The Most Enriched GO Terms(miRNA vs Wheat)

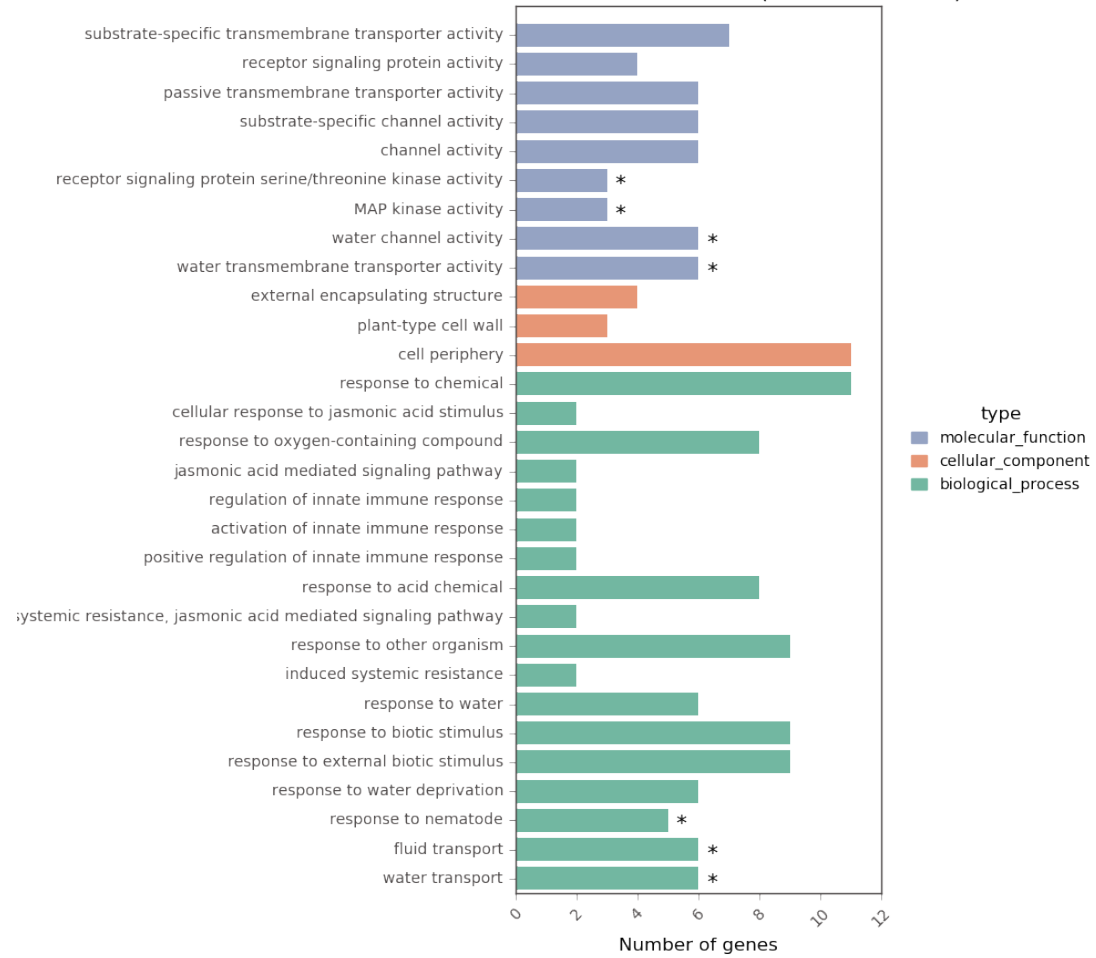

Supplement: S3 Fig — (PDF) [file pone.0175924.s003.pdf]

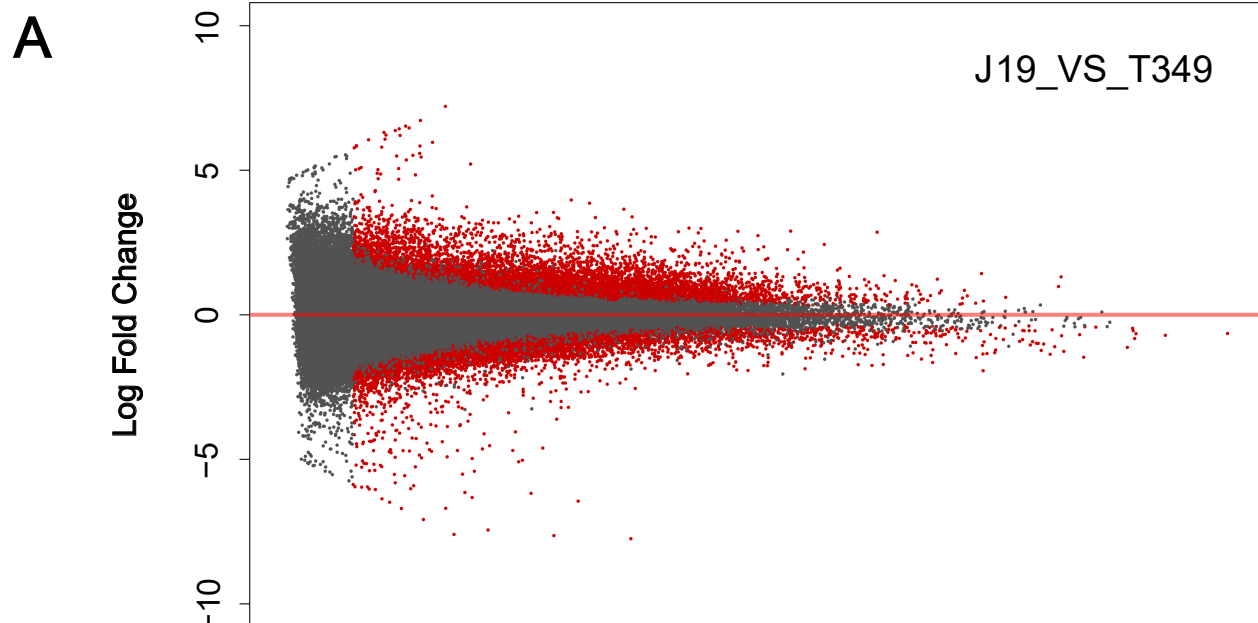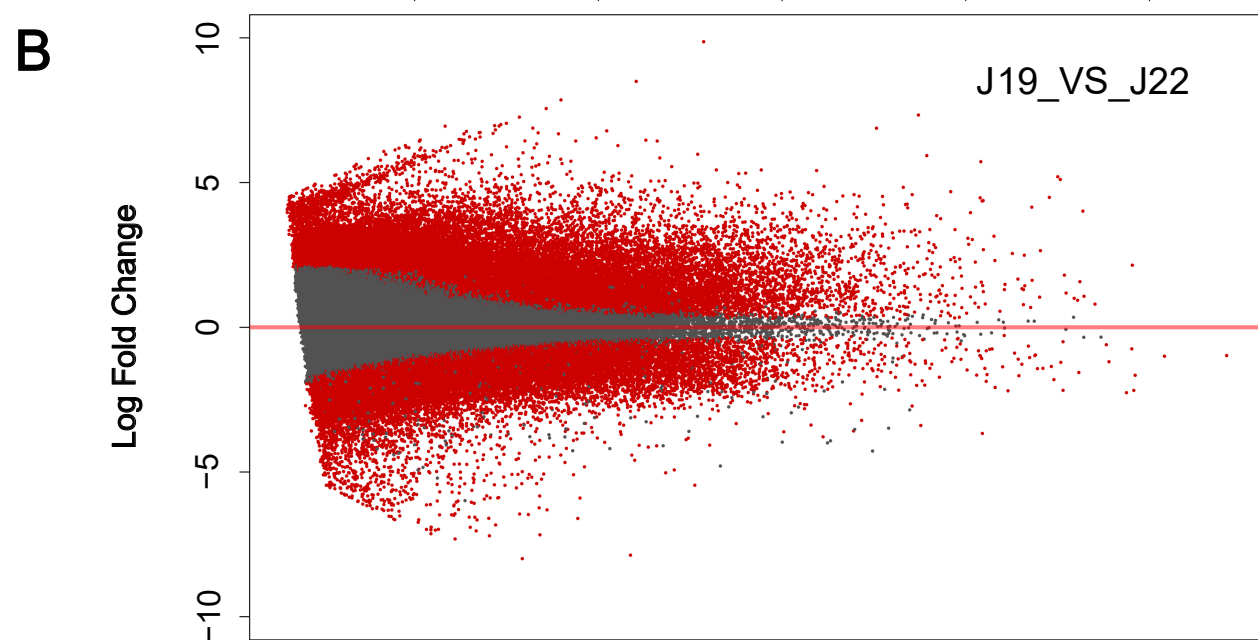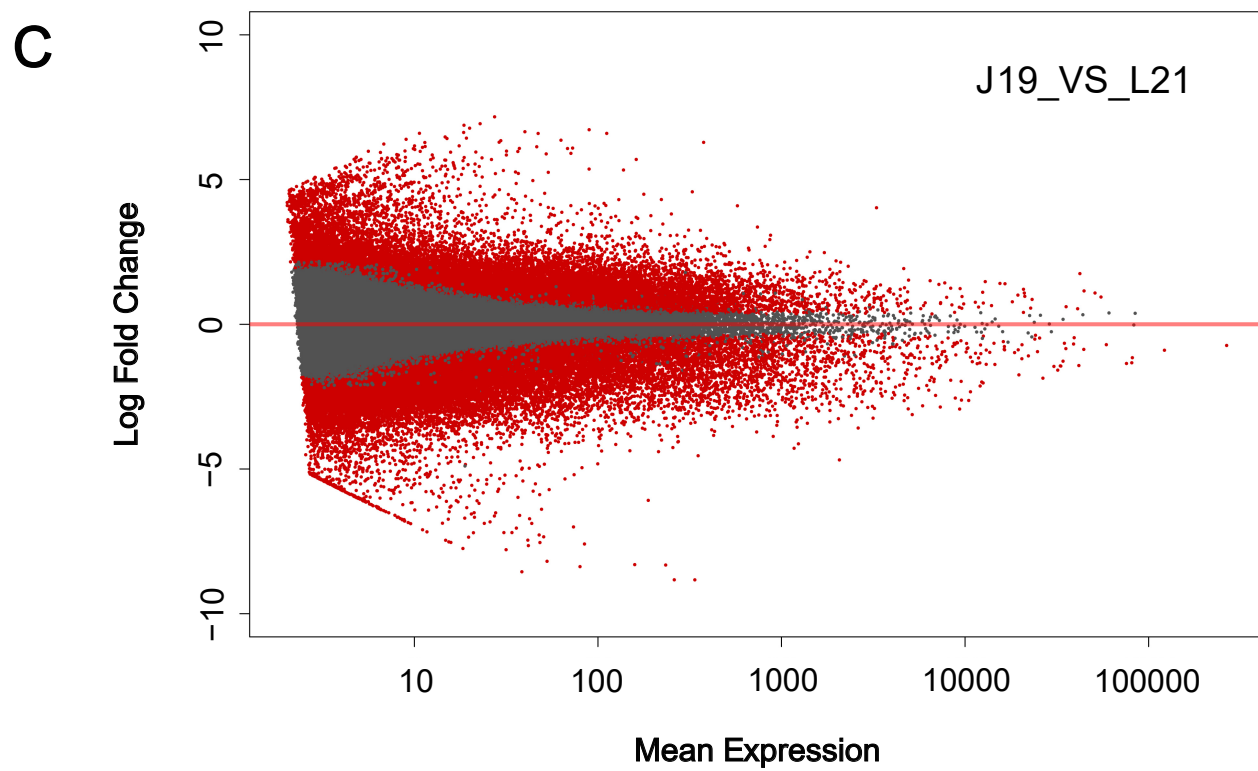

Supplement: S4 Fig — (PDF) [file pone.0175924.s004.pdf]

A

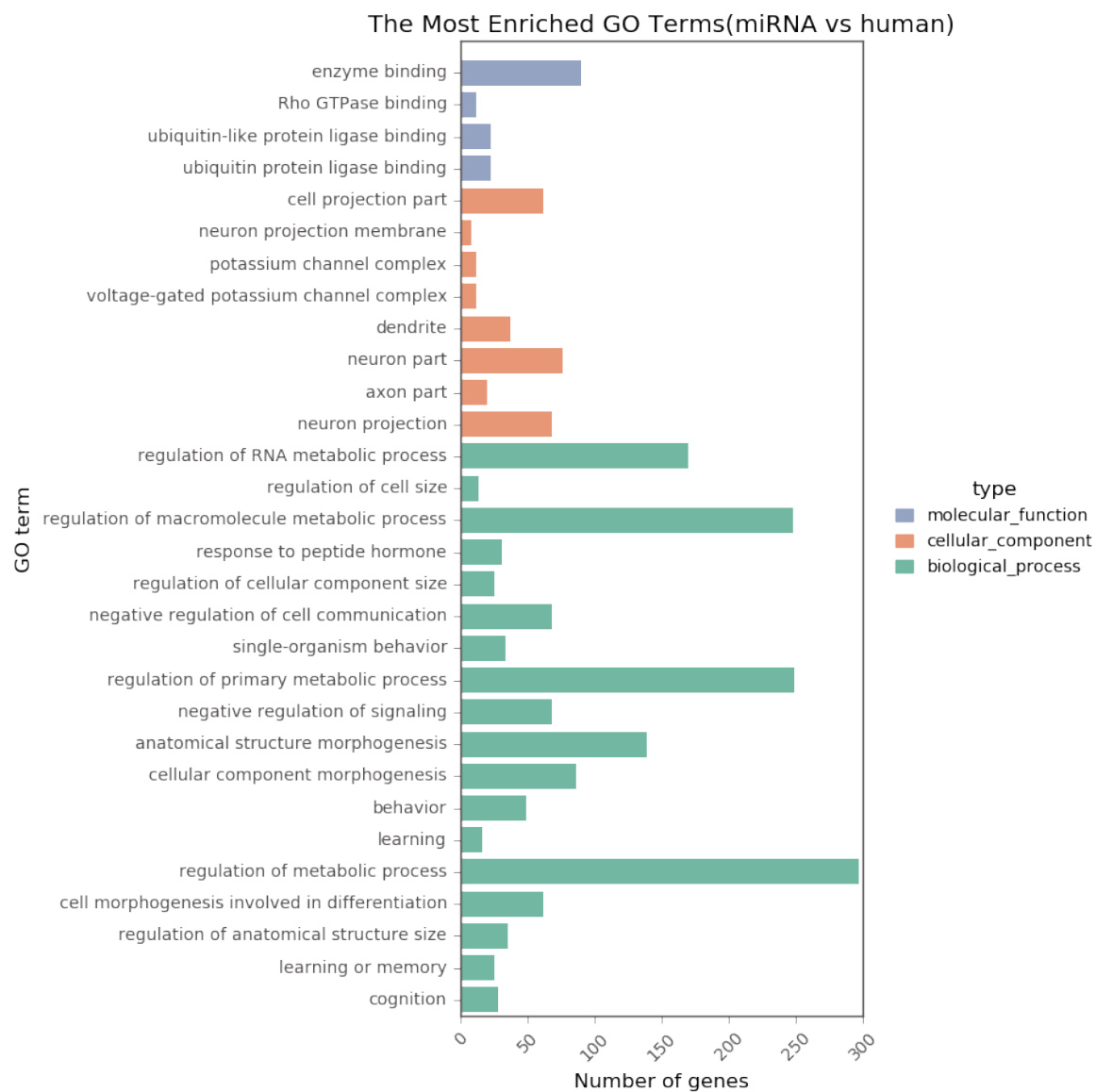

B

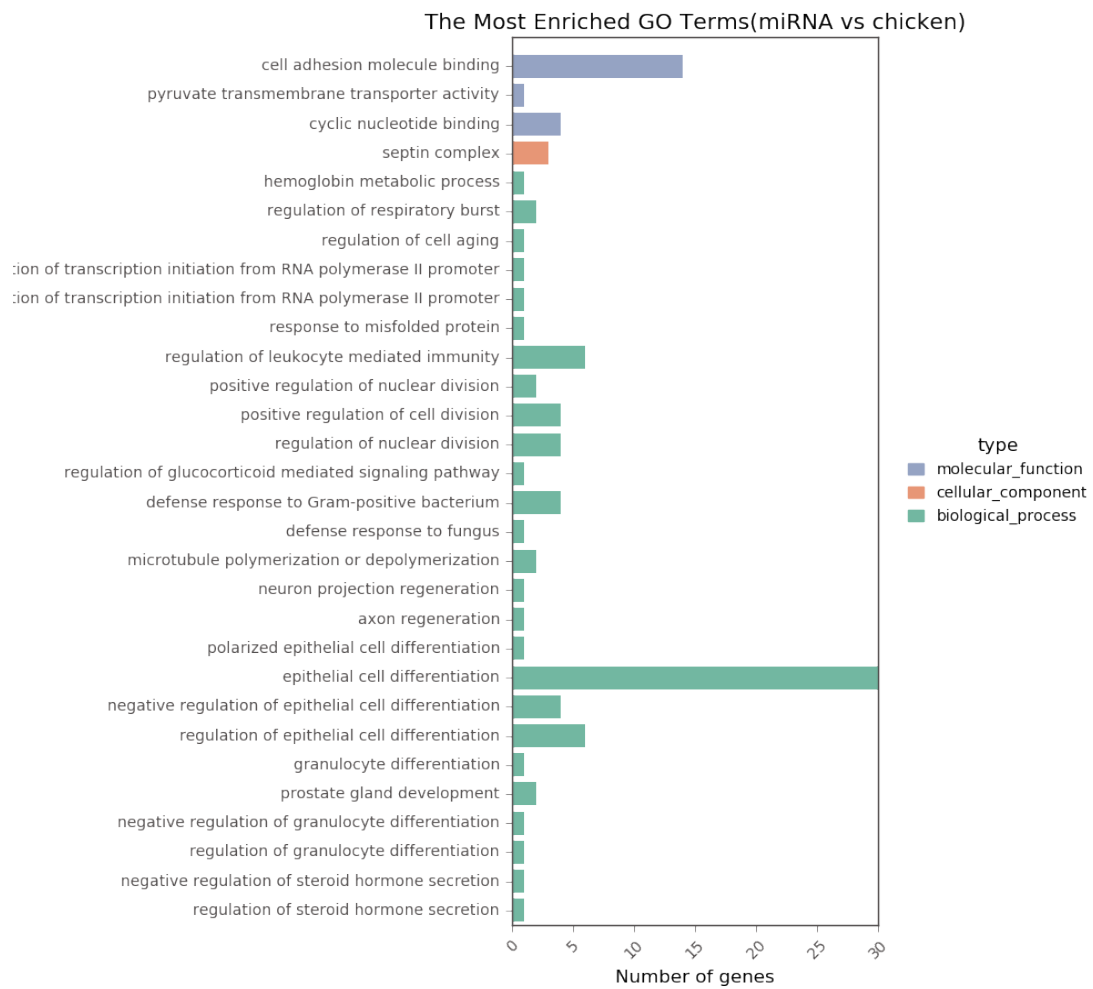

Supplement: S5 Fig — (PDF) [file pone.0175924.s005.pdf]

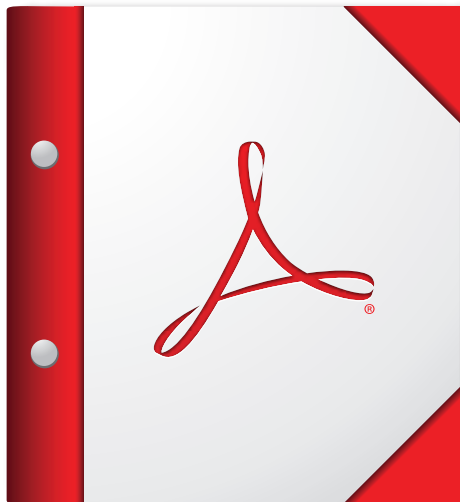

为获得最佳体验，请在 **Acrobat 9、Adobe Reader 9**  
或更高版本中打开此 **PDF** 包。

[立即购买 Adobe Reader !](#)

Supplement: S2 File — (PDF) [file pone.0175924.s007.pdf]
